# Safety and Efficacy of Low-Dose Triamcinolone Injection without Injection Frequency Limitation for Trigger Finger

**DOI:** 10.1016/j.jhsg.2025.01.005

**Published:** 2025-02-05

**Authors:** Jo Watanabe, Yusuke Matsuura, Takahiro Yamazaki, Toshiyuki Yamada, Seiji Ohtori

**Affiliations:** ∗ Department of Orthopaedic Surgery, Matsudo City General Hospital, Matsudo, Chiba, Japan; † Department of Orthopaedic Surgery, Graduate School of Medicine, Chiba University, Chuo-ku, Chiba, Japan; ‡ Department of Orthopaedic Surgery, Eastern Chiba Medical Center, Togane, Chiba, Japan; § Department of Orthopaedic Surgery, Chiba Aoba Municipa Hospital, Chuo-ku, Chiba, Japan

**Keywords:** Steroid injections, Trigger finger

## Abstract

**Purpose:**

Triamcinolone tendon sheath injection is a useful nonsurgical treatment for trigger finger; however, complications, such as tendon rupture, and infections caused by excessive administration, have been reported. Considering the complication risk, we inject 4 mg triamcinolone into the tendon sheath without limitation on the number of injections at intervals of at least 1 month. This study aimed to retrospectively examine the results of triamcinolone tendon sheath injections for trigger finger at multiple facilities.

**Methods:**

The participants included patients with trigger finger who visited four facilities between April 2009 and October 2021, received at least one triamcinolone tendon sheath injection, and could be evaluated for effectiveness. Patients with a follow-up period of <3 months from the initial injection, and pediatric patients aged <16 years old were excluded. Quinnell’s severity classification, number of injections per finger, interval of injections (when performed ≥2 times), complications, proportion of diabetes, hemoglobin A1c (HbA1c) levels, and proportion that required surgery were collected.

**Results:**

Overall, 356 cases and 715 fingers were included (men, 260 fingers; women, 455 fingers). The mean age of the participants was 64.9 years (17–92 years), and the mean number of affected fingers per person was 1.9 (1–7 fingers). The median follow-up period was 27 (3–134) months. According to Quinnell’s severity classification, 234, 274, 126, and 50 fingers were classified as grade 1, 2, 3, and 4, respectively. The average number of injections per finger was 3.3. The mean interval between injections was 6.3 months. Complications, such as tendon rupture, or infection, were not observed. The rate of diabetes was 22.4%, and the median HbA1c value was 7.2%. In total, 9.8% of all patients required surgery.

**Conclusions:**

No complications were observed for 4 mg triamcinolone injections when administered at intervals of at least 1 month.

**Type of study/level of evidence:**

Therapeutic Ⅳ.

Trigger finger is a common condition with a lifetime incidence of 2% to 3%.^1^ Triamcinolone acetonide (TA) injection into the tendon sheath is one of the nonsurgical treatments and is useful as an initial treatment for trigger finger.^2^ Complications such as tendon rupture, tendon sheath rupture, and infection related to excessive administration of TA have been reported.^3^, ^4^, ^5^, ^6^, ^7^, ^8^, ^9^, ^10^ Thus, a discussion regarding the number of doses, frequency, and dosage is warranted. Regarding the dosage, all reports of complications involved a TA dose of ≥10 mg.

Kerrigan et al^11^ reported that limiting the number of preoperative injections to three is the best in terms of treatment cost. A previous review recommended a maximum number of thee injections at any one site.^12^ Brozovich et al^13^ also recommended limiting the number of injections to two for a single trigger thumb in women without diabetes mellitus (DM) and immediate percutaneous tendon sheath release for other patients. However, surgical complications, such as neurovascular injury, and wound problems do occur.^14^, ^15^, ^16^, ^17^, ^18^ We also observed that patients preferred nonsurgical treatment. This included patients who do not wish to undergo surgery after being explained the effects and complications of the surgery, patients who are busy with work or family care and find it difficult to take time off, and patients in medically underserved areas where access to surgery is challenging.

Therefore, to avoid surgery, we performed tendon sheath injections without frequency limitations according to the patient’s wishes. The injection dose was 4 mg TA with an interval of at least 1 month. TA was selected because it has higher initial improvement rates and lower rates of progression to surgery compared to dexamethasone.^19^^,^^20^

This study investigated the clinical outcomes of patients with trigger finger who received steroid injections.

## Materials and Methods

This study was approved by the Ethics Committee of the Graduate School of Medicine, Chiba University (approval no. 10474). Of the 1,472 cases and 1,936 fingers diagnosed with trigger finger at four related facilities between April 2009 and October 2021, this study analyzed 356 cases and 715 fingers that received TA tendon sheath injections and could be evaluated for effectiveness.

Tendon sheath injections for trigger finger are performed by multiple physicians who conduct specialized outpatient hand surgery at each facility. Injections were indicated for cases with tenderness, snapping, or locking at the A1 pulley site that did not improve with rehabilitation (A1 pulley stretching) or medication.^21^^,^^22^ The effectiveness of tendon sheath injection was evaluated based on the medical records at the time of the visit after the injection. Patients who did not return after the tendon sheath injection, patients with a follow-up period of <3 months from the initial injection, and pediatric patients aged <16 years were excluded. The injection solution was a mixture of 1 mL of 1% lidocaine and 4 mg (0.1 mL) of TA (KENACORT-A Intramuscular Intraarticular, Bristol Myers Squibb) to yield 1.1 mL total. Tendon sheath injection is performed under direct vision using a 27-G injection needle from above the A1 pulley or on the side of the A2 pulley. Regarding the injection, the procedure was either performed under ultrasound guidance, or extratemdinous injection was performed if resistance was believed to avoid intratendinous injection.^23^ Extratemdinous injection was considered acceptable because of comparable therapeutic outcomes between extratemdinous and intratendinous steroid injections for trigger finger.^24^ Either of these two approaches were selected at the physician’s discretion based on individual cases and clinical findings. Additionally, Oh et al^25^ reported a case of flexor tendon sheath rupture occurring because of excessive gripping 1 week after the injection. Therefore, patients were instructed to avoid excessive gripping for approximately 1 week immediately after the injection.

The interval between administrations was at least 1 month, and second and subsequent injections were indicated for cases with residual pain, snapping, or locking. We thoroughly explained the risk of complication, such as tendon rupture and infection, and that surgery is typically recommended after 2–3 injections. However, for patients who still preferred injection despite this information, we continued to provide the injections without frequency limitations. For patients with DM, the risk of increased blood glucose levels was also explained. Postinjection rehabilitation involved each physician instructing the patient to perform A1 pulley stretching.^21^^,^^22^

Surgery was indicated for cases where pain had improved after tendon sheath injection but snapping or locking persisted and cases where the patient wished to undergo surgery because of recurrence despite multiple injections. For patients with pain only and in cases where pain did not improve despite injections, surgery was performed if the patient desired it. Whether to perform percutaneous tendon sheath release or open tendon sheath release was at the discretion of the surgeon.

Cases were graded according to Quinnell’s severity classification: grade 1, tenderness at the A1 pulley only; grade 2, snapping but no locking; grade 3, snapping with locking; and grade 4, joint contracture.^26^ Quinnell’s severity classification was evaluated by the physician who conducted the initial examination, and where assessment was possible, cases were extracted based on medical records. Past medical records were reviewed to determine the total number of injections per finger, injection interval (only for cases with ≥2 injections), DM as a comorbidity, hemoglobin A1C (HbA1c) value at the time of the first injection, the proportion that required surgery, number of injections until surgery, and complications. The surgery rate, proportion with only one injection (one-injection rate), average number of injections (excluding cases with only one injection), and average injection interval were also examined by grade. For DM, the surgery and recurrence rates (proportion that received multiple injections) were also examined by dividing patients into the non-DM and DM groups.

For statistical analysis, the chi-square test was used for the analysis of the surgery rate and one-injection rate by grade, the Kruskal–Wallis test was used for the number of injections and injection interval, and the chi-square test for the surgery rate and recurrence rate by the presence or absence of DM. A *P* value of <.05 was considered significant.

## Results

In total, 356 cases and 715 fingers were included in the study, including 260 fingers in males and 455 fingers in females, with a mean age of 64.9 (17–92) years. The median follow-up period from the initial injection was 27 (3–134) months, and the mean number of affected fingers per person was 1.9 (1–7) fingers. There were 421 right fingers and 294 left fingers, including 175 thumbs, 96 index fingers, 243 middle fingers, 155 ring fingers, and 46 little fingers.

**Figure 1:**
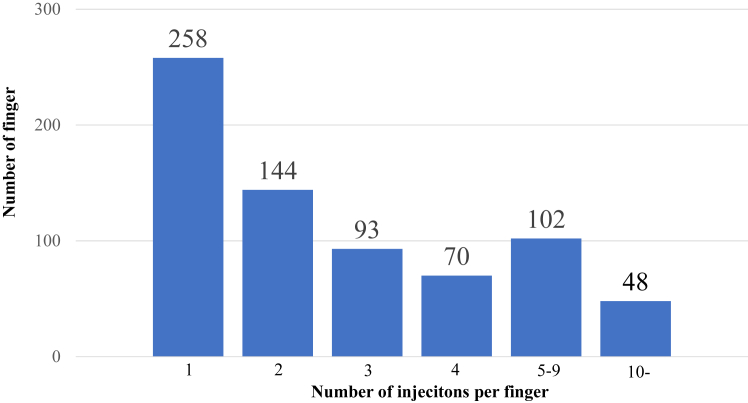
Number of injections per finger. The average was 3.3, and 457 fingers received multiple injections.

**Figure 2:**
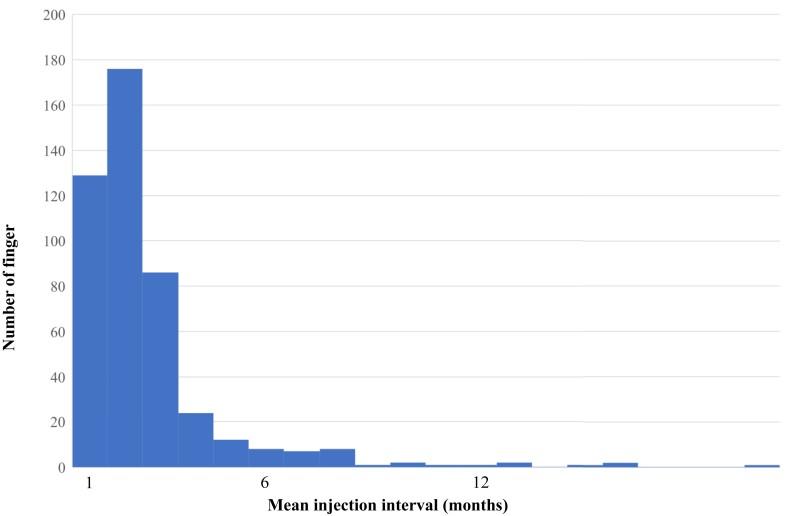
Mean injection interval. The mean was 4.9 months; 99% were within 1 year.

Of the 715 cases, 684 cases had information on Quinnell’s severity classification based on medical their records. In total, 234 fingers had grade 1, 274 had grade 2, 126 had grade 3, and 50 had grade 4 severities. The mean number of injections up to the point when patients underwent surgery or for those who were lost to follow-up was 3.3; this distribution is shown in Figure 1. The symptoms disappeared with one injection in 258 fingers, which was the most common, and 457 fingers had multiple injections, which accounted for more than half of the cases. Moreover, 48 fingers received ≥10 injections. The median interval between injections was 4.9 months. Injection intervals of 1 month were performed in 129 digits and 2 months in 176 digits, accounting for 67% of all cases. The injection interval was <1 year in 99% of the cases, and an interval of ≥1 year was recorded in six fingers (Fig. 2).

The DM comorbidity rate was 22.4% (80 cases, 203 fingers). In 74 out of 80 cases in which it could be evaluated, the median HbA1c was 7.2% (5.6% to 13%). The mean number of injections per finger for patients with diabetes was 3.6.

The overall surgery rate was 9.8% (51 cases, 70 fingers), of which 20% (9 cases, 14 fingers) underwent percutaneous release surgery. The surgery rate for patients with diabetes was 15.3% (11 cases, 181 fingers), of which 11.1% (2 cases, 2 fingers) underwent percutaneous release surgery. The mean number of injections until surgery was 3.5 (1–11). No complications were observed in any case.

**Figure 3:**
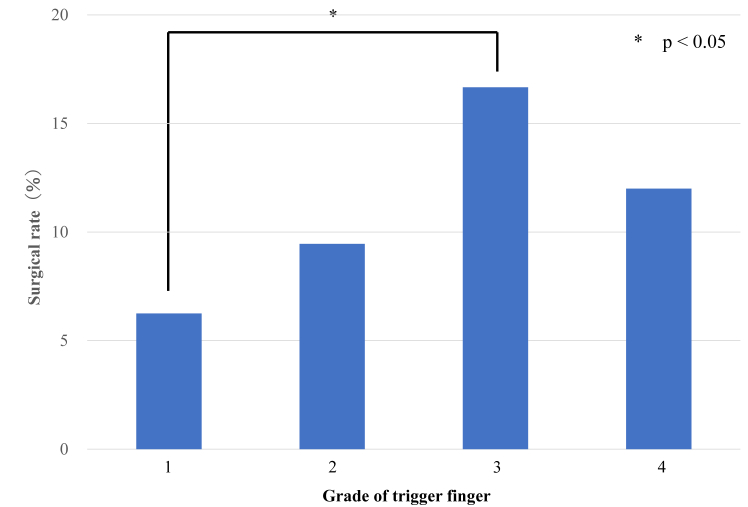
Surgical rate by grade. Grades 1 and 3 showed a significant difference.

**Figure 4:**
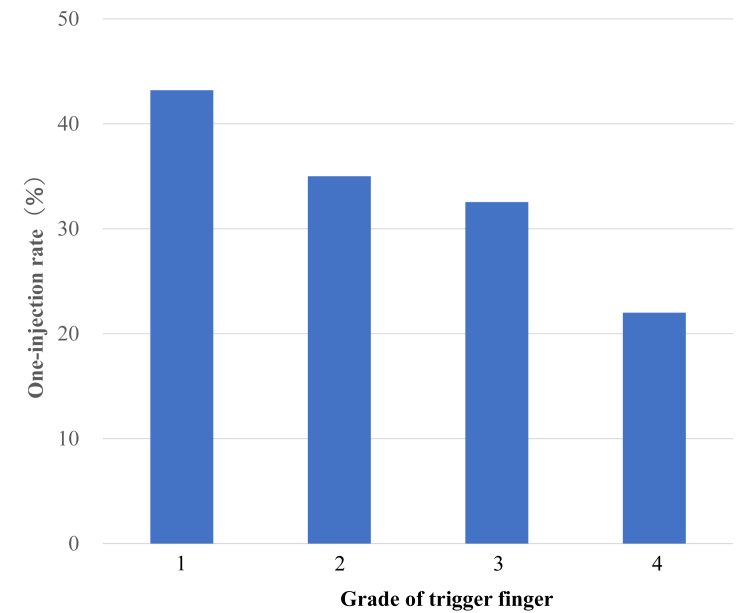
One-injection rate by grade. This tended to decrease as the grade increased; however, no statistically significant differences were found.

**Figure 5:**
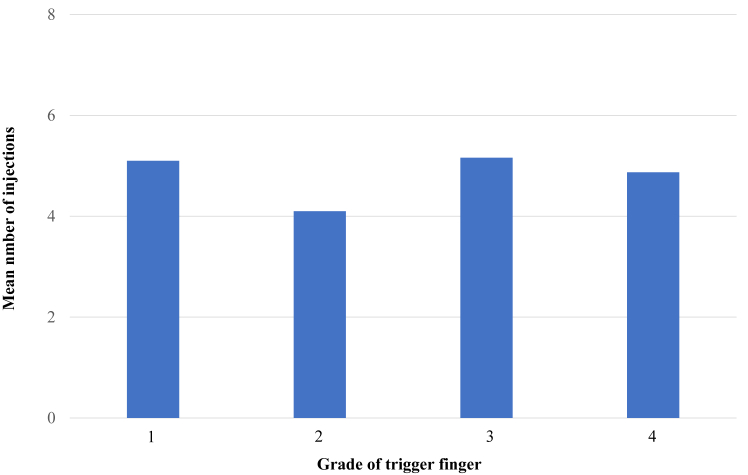
Mean number of injections by grade. No statistically significant differences were observed.

**Figure 6:**
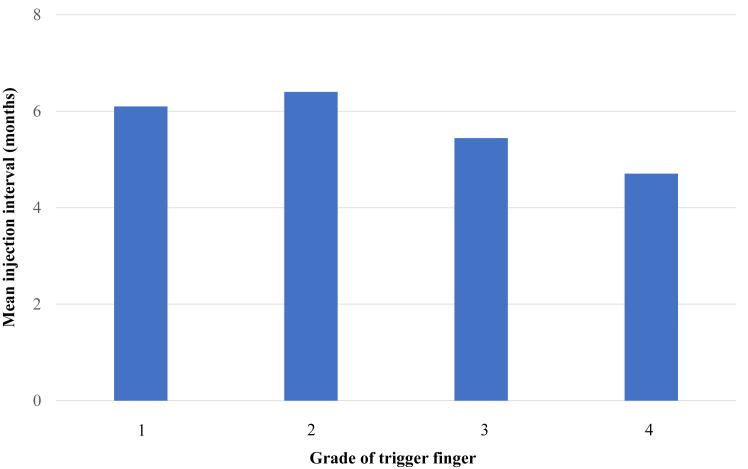
Mean injection interval by grade. No statistically significant differences were observed.

When examined by grade, the surgery rates were 6.4%, 9.5%, 16.7%, and 12% for grades 1, 2, 3, and 4, respectively, with a significant difference between grades 1 and 3 (Fig. 3). The one-injection rate was 43.2% for grade 1, 35% for grade 2, 32.5% for grade 3, and 22% for grade 4, with no significant difference (Fig. 4). The mean number of injections, excluding cases with only one injection, was 5.1 for grade 1, 4.1 for grade 2, 5.2 for grade 3, and 4.9 for grade 4, with no significant difference (Fig. 5). The mean injection interval was 6.1 months for grade 1, 6.4 months for grade 2, 5.4 months for grade 3, and 4.7 months for grade 4, with no significant difference 3 (Fig. 6).

Regarding DM, the surgery rate was 11.3% (58 of 512 fingers) in the non-DM group and 8.8% (18 of 203 fingers) in the DM group, with no significant difference. The recurrence rate was 61.9% (317 of 512 fingers) in the non-DM group and 68.5% (139 of 203 fingers) in the DM group, with no significant difference.

## Discussion

In this study, tendon sheath injections were performed with 4 mg of TA at an interval of at least 1 month and with a median interval of 4.9 months, and complications were not observed even with multiple injections. Six studies have evaluated multiple tendon sheath injections using TA (Table 1).^2^^,^^27^, ^28^, ^29^, ^30^, ^31^ To the best of our knowledge, this study had the largest number of cases. The mean age in this study was 64.9 years, similar to other reports. Regarding the TA dose, the present study used 4 mg. Freiberg et al^27^ reported using 1–3 mg. Schubert et al^31^ reported using 8 mg. Maneerit et al^28^ and Dala-ali et al^30^ reported using 10 mg. Marks et al^2^ reported using 16 mg. Rozental et al^29^ reported using 40 mg. Many studies were using TA doses higher than used in the present study. The mean number of injections was 3.3 in this study, which was higher than the reports by Freiberg et al^27^ (1.2), Marks et al^2^ (1.1), Maneerit et al^28^ (1.2), Rozental et al^29^ (1.2), Dala-ali et al^30^ (2.1), and Schubert et al^31^ (1.3). The surgery rate was 9.7% in the present study, which was nearly the same as that reported by Marks et al^2^ (9%) and lower than that reported by Freiberg et al^27^ (32.7%), Maneerit et al^28^ (13.3%), Rozental et al^29^ (22.6%), Dala-ali et al^30^ (34%), and Schubert et al^31^ (20%).

**Table 1:** Comparison of Reports that Performed Multiple TA Tendon Sheath Injections

Authors	n	Mean age (y)	TA dose (mg)	Mean number of injections	Mean interval injection (mo)	Surgical rate (%)	Complication	Year published
Freiberg et al^27^	101	57.4	1–3	1.2 (1–3)	3	32.7	None	1989
This study	715	64.9	4	3.3 (1–23)	4.9	9.8	None	2024
Schubert et al^31^	577	59.5	8	1.3 (1–4)	10.4	20	None	2013
Maneerit et al^28^ (only thumb)	60	53	10	1.2 (1–3)	Unknown	13.3	Cellulitis 1 case	2003
Dala-ali et al^30^	90	62	10	2.1 (1–3)	Unknown	34	None	2012
Marks et al^2^	74	56	16	1.1 (1–2)	37	9	None	1989
Rozental et al^29^	124	62.3	40	1.2 (1–2)	Unknown	22.6	None	2008

In this study, despite the low TA dose compared with other reports, the surgery rate was low. However, direct comparison of surgical rates between this study and others was difficult because of the differences in injection frequency limitation and surgical indication criteria. As far as we could ascertain from our literature review, seven cases and nine fingers were observed to have complications after tendon sheath injection where the TA dose was specified (Table 2).^3^, ^4^, ^5^, ^6^, ^7^, ^8^ Cases with TA dose of ≥20 mg accounted for the majority, with tendon sheath rupture in three fingers and flexor tendon rupture in 6.^3^, ^4^, ^5^, ^6^, ^7^, ^8^, ^9^ The number of injections ranged widely from 1 to 30 times, with most cases occurring within 6 months. Several of these studies have reported special findings of the tendon and tendon sheath during surgery. Nanno et al^5^ reported histological findings of decreased viable fibroblasts around the degenerated tendon fibers, partial neovascularization, and presence of mild inflammation. Gyuricza et al^8^ reported that the flexor tendon sheath was gray with evidence of chronic inflammation, and complete destruction of the pulleys from A1 to A4 was observed. Hamano et al^9^ confirmed complete rupture of the pulleys from A1 to A4 and reported that the flexor digitorum superficialis tendon was detached from its insertion at the base of the middle phalanx, whereas the flexor tendon of the middle finger was surrounded by fibrous scar tissue.

**Table 2:** Complications of TA Tendon Sheath Injection

Authors	Sex	Age (y)	TA dose (mg)	Number of injections	Mean interval injection (mo)	Onset (mo)	Complication	Site	Year published
Nanno et al^5^	F	56	10	2	Unknown	2	Tendon rupture	FPL	2014
Gyuricza et al^8^	F	42	20	3	6	5	Tendon sheath rupture	Middle finger	2009
Yamada et al ^4^	F	37	20	7	2	2	Tendon rupture	FDP of little finger	2011
Hamano et al^9^	F	40	20	30	1	Unknown	Tendon sheath rupture	Middle/Ring finger	2013
Inomori et al^7^	F	56	20	5	7.2	6	Tendon rupture	FPL	2023
Fitzgerald et al^3^	M	77	40	2	4.5	6	Tendon rupture	FDP/FDS of middle finger	2005
Tanaka et al^6^	M	45	40	1	-	3	Tendon rupture	FDP/FDS of little finger	2017
Tanaka et al^6^	F	57	40	2	2	2	Tendon rupture	FPL	2017

NOTE. Only reports with known TA dose.

Abbreviations: FDP, flexor digitorum profundus; FDS, flexor digitorum superficial.

Wong et al^32^ reported that TA decreases tendon cell viability in a concentration-dependent manner, and the TA dose may be a factor in tendon rupture. The lowest TA dose reported was 10 mg.^5^ However, in our review of the literature, no studies have reported complications of tendon sheath injection of 4 mg of TA for trigger finger.

Bookman et al^33^ examined the treatment results of different TA doses (5 mg vs 10 mg vs 20 mg), and they found no significant difference in visual analog scale scores or *Quick*DASH (Disabilities of the Arm, Shoulder, and Hand) questionnaire scores between different doses. From these findings, the TA dose of 4 mg may be able to achieve sufficient therapeutic effect without causing complications.

In Japan, the package insert for TA states that the interval between administrations should be at least 2 weeks. Studies have reported that the survival rate of rotator cuff-derived cells and the strength of rat Achilles tendons improve 3 weeks after TA administration, and that trigger finger symptoms improve within 30 days in most cases with 5 mg TA injection.^34^, ^35^, ^36^ Based on these findings, an interval of at least 1 month is considered desirable to evaluate the effect of TA injection and consider the next injection. However, as shown in Table 2, complications occurred even with an interval of >1 month between administrations; thus, the dose should be given more importance than the interval between administrations to avoid complications.

In this study, the results were also examined by grade. Although the surgery rate appeared to increase with higher grades, exhibiting a significant difference between grades 1 and 3, the actual decision to proceed with surgery was primarily based on patient preference, even in cases with persistent triggering or locking despite pain relief after injection. Although not significant, the one-injection rate tended to decrease as the grade increased. The proportion of cases requiring multiple injections may increase as the severity increases. Therefore, recommending surgery as the grade increases is appropriate. However, even in grade 3, which has the highest surgery rate, the one-injection rate is 32.5%. Given the possibility of improvement with injection, attempting one injection before proceeding to surgery is reasonable.

This study included DM cases, but there were no infection cases. There was no significant difference in the surgery rate and recurrence rate between the DM group and the non-DM group. DM cases generally have an increased risk of recurrence after trigger finger injection; accordingly, more cases become indicated for surgery.^28^ However, DM is one of the risk factors for postoperative infection; thus, there may be more cases that avoid surgery, resulting in a lack of difference in the surgery rate.^14^

This study has three major limitations. First, multiple physicians perform the injections. There may be inconsistencies in medications, rehabilitation, follow-up period, indications for injection in DM, and indications for surgery. Second, when recurrence, or complications occur, patients may not necessarily visit the affiliated hospitals and may receive treatment at other hospitals. No protocol was established to contact patients who did not complete follow-up, and such events could not be confirmed. Third, we were unable to track the blood glucose levels of patients with diabetes after trigger finger injection. Therefore, the injection may have caused deterioration in blood glucose control in patients with diabetes. Several studies have reported on blood glucose trends following steroid injections. Sotani-Ogawa et al^37^ reported no significant difference in blood glucose trends after injection of 20 mg TA for macular degeneration in diabetic patients. Kaderli et al^38^ stated no significant difference in blood glucose control or adrenal function in a comparative study of 40 mg TA injection versus saline in patients with diabetes. Conversely, Alexander et al^39^ reported that for subacromial steroid injections, patients with poor blood glucose control (HbA1c ≥ 7) or insulin-dependent diabetes showed significant increases in blood glucose levels.

Based on these findings, blood glucose trends should be monitored after steroid injections for trigger finger, particularly among patients with poor blood glucose control or those using insulin. However, to the best of our knowledge, no studies have reported on blood glucose trends following TA injections for trigger finger, and further research is awaited.

In conclusion, tendon sheath injection with 4 mg TA is likely to avoid complications even with multiple injections if an interval of at least 1 month is maintained. To avoid complications, the dose should be given more importance than the interval between administrations. It is considered a useful method for cases where surgery is difficult or nonsurgical treatment is desired.

## Conflicts of Interest

No benefits in any form have been received or will be received related directly to this article.
